# P275: Prevention of healthcare-associated infections associated with care: results of the neonatal service audit in Beni-Messous university hospital Algiers in 2012

**DOI:** 10.1186/2047-2994-2-S1-P275

**Published:** 2013-06-20

**Authors:** G Brahimi, S Mounes, R Belkaid, A Soukehal

**Affiliations:** 1 Department of Epidemiology and Preventive Medicine, CHU Béni-Messous, Algiers, Algeria

## Introduction

The prevention of the risk of contamination of the newborn environment requires strict organization of care.

## Objectives

Evaluate adherence and compliance to maintenance of incubators and adherence to hand hygiene (HH) and good practices related to the installation of peripheral venous catheters (PVCs).

## Methods

The audit took place from 1 to 31 March 2012 in the neonatology unit by questioning and observing the paramedical personnel.

## Results

The unit has 35 beds (20 incubators and 15 bassinets), the 20 caregivers were audited, 70% nursery and 20% of graduated nurses.

Daily cleaning of the inside of the incubator was made in 80% of cases, the disinfectant spray was used in 25%. Cleaning the outside of the incubator was performed with water in 81.6%. Of 105 treatments performed, the HH compliance rate before and after the procedure is 62.8%. Adequacy in both opportunity and type of HH was found in 54.3%. When installing a PVC, the cleaning phase was observed in 15% of cases, with no rinsing or drying. No healthcare worker respected the time need for antiseptic drying. Sterile gauzes are used in 55% of cases. Gloves were used for the placement of a PVC in 20%.

## Conclusion

The risk of infection related to the maintenance procedures of incubators and gestures of care for the newborn is identified; it could be avoided by the application of appropriate and validated procedures.

## Competing interests

None declared

